# Hemoglobin levels during the first trimester of pregnancy are associated with the risk of gestational diabetes mellitus, pre-eclampsia and preterm birth in Chinese women: a retrospective study

**DOI:** 10.1186/s12884-018-1800-7

**Published:** 2018-06-26

**Authors:** Chen Wang, Li Lin, Rina Su, Weiwei Zhu, Yumei Wei, Jie Yan, Hui Feng, Boya Li, Shuang Li, Huixia Yang

**Affiliations:** 1 Department of Obstetrics and Gynecology of Peking University First Hospital, Xianmen Street No. 1, Xicheng District, Beijing, 100034 China; 2 National Institute of Hospital Administration, Beijing, China

**Keywords:** Hemoglobin, Gestational diabetes mellitus, Pre-eclampsia, Preterm birth, Body mass index

## Abstract

**Background:**

Hemoglobin (Hb) measurement is a standard test among pregnant women during the first perinatal visit that is used to evaluate physical status and anemia. However, studies focusing on Hb levels and pregnancy outcomes are scarce. This study aimed to determine whether Hb levels in early pregnancy were associated with the risk of gestational diabetes mellitus (GDM), pre-eclampsia (PE) and preterm birth.

**Methods:**

A hospital-based retrospective study was conducted among 21,577 singleton, non-smoking pregnancies between June 2013 and January 2015. The demographic data and medical information of each participant were collected individually through questionnaires and patient medical records. Odds ratios were generated using a multivariate logistic regression analysis to evaluate the relative risk of GDM, PE and preterm birth continuously and across different hemoglobin ranges in the overall population and in women from different pre-pregnancy body mass index (BMI) categories, respectively. The level of statistical significance was set at 0.05.

**Results:**

(1) For women who were underweight, normal-weight, overweight and obese, early pregnancy Hb levels were 127.8 ± 10.1 g/L, 129.6 ± 9.7 g/L, 132.2 ± 9.5 g/L and 133.4 ± 9.4 g/L, respectively. (2) Women with GDM and PE had significantly increased Hb levels during early pregnancy compared with controls, whereas women with preterm birth processed significantly decreased Hb levels. (3) After adjusting for confounders, the risks for GDM and PE increased with high maternal Hb (OR: 1.27 for Hb 130–149; OR: 2.06 for Hb ≥ 150 g/L), and the risk for preterm birth decreased with high maternal Hb (OR: 1.30 for Hb 130–149; OR: 2.38 for Hb ≥ 150 g/L) and increased with low maternal Hb (OR: 1.41 for Hb <  110 g/L). Among women whose BMI was < 24 kg/m^2^, high GDM (OR: 1.27 for Hb 130–149; OR: 1.84 for Hb ≥ 150 g/L) and low preterm rates (OR: 0.77 for Hb 130–149; OR: 0.23 for Hb ≥ 150 g/L) were observed with high Hb, whereas in women whose BMI was ≥24 kg/m^2^, only high GDM rates were observed with Hb > 150 g/L (OR: 2.33).

**Conclusion:**

These findings suggest that Hb levels during early pregnancy play a role in predicting the risk of GDM, PE and preterm birth.

## Background

Gestational diabetes mellitus (GDM) and pre-eclampsia (PE) are two of the most common complications during pregnancy. Both GDM and PE contribute significantly to maternal, fetal and neonatal morbidity and mortality. Furthermore, they may result in adverse consequences for the health of both the mother and offspring later in life [1–3]. Previous epidemiological studies have shown that GDM and PE occur in 9.3%–25.5% [4] and 0.2–9.2% [3] of pregnancies, respectively, in different global populations. Additionally, preterm birth, which accounts for 11.1% of live births worldwide, is also an important concern during pregnancy [5]. Preterm birth is a major determinant of neonatal death or child loss. Furthermore, children who are born prematurely have a higher risk of cerebral palsy, cardiovascular disease, pulmonary illnesses or psychological diseases, and these conditions can persist throughout life [6]. Thus, predicting GDM, PE, and preterm birth is essential and imperative for improving the health quality of populations.

Hemoglobin (Hb) measurement is a standard test among pregnant women during the first perinatal visit that is used to evaluate physical status and anemia. According to the World Health Organization [7], anemia is diagnosed when a blood test shows an Hb value of less than 110 g/L in pregnant women. Observational studies have found that anemia during pregnancy is associated with detrimental pregnancy outcomes, including preterm birth, low birth weight, infection and hemorrhage [8]. Additionally, several studies have even reported that high Hb levels during pregnancy could also be a predictor or cause of some pregnancy complications [9–11]. However, studies focusing on Hb levels and pregnancy outcomes are scarce, and the findings are inconsistent due to a wide variation in study designs, sample sizes, populations and the time of Hb testing. Moreover, high maternal Hb has not received the same attention as anemia because it is more likely to be perceived as a symbol of good nutrition status. Furthermore, previous studies indicated that Hb levels during pregnancy were significantly associated with body mass index (BMI) [12]. However, few studies to date have assessed the effects of maternal Hb values in the first trimester stratified by pre-pregnancy BMI on pregnancy complications. Thus, in this large sample study, we aimed to conduct stratified analyses modified by pre-pregnancy BMI and to evaluate whether associations exist between Hb levels in the first trimester and the risk of GDM, PE, and preterm birth.

## Methods

### Study design and participants

Data for this current analysis was obtained from a hospital-based retrospective study of 44,002 pregnant women who gave birth between June 2013 and January 2015 at 21 hospitals throughout three cities in China. One of the three cities was Beijing, the capital city of China, which is located in northern China. The two other cities were Guangzhou and Chengdu, which are the provincial capital cities of Guangdong and Sichuan provinces, located in southern and western China, respectively. Participants in our study were stratified according to their Hb levels in the first trimester combined with their pre-pregnancy BMI. These values were compared to the incidence rates of GDM, PE, and preterm delivery. The study was reviewed and approved by the Institutional Review Board of the First Hospital, Peking University (Reference number: 2013[578]). All participants provided written informed consent, and the Ethics Committee approved this consent procedure.

All participants in the retrospective study were eligible for the present analysis if they were singleton, non-smoking pregnant women with their Hb measured at < 14 weeks of gestation and had complete maternal and infant records. However, if the participants in our study were reported a diagnosis of diabetes, chronic hypertension, cardiovascular disease, thyroid disorder, respiratory ailments, placenta previa, fetal anomalies or histories of poor pregnancy outcomes, they would be excluded. Overall, a total of 21,577 participants were available for and included in the final analysis.

### Data collection

The demographic and medical information for each participant were collected by using a structured questionnaire the day after they gave birth. Demographic information, such as maternal age, pre-pregnancy weight, height, educational level and family financial status were collected from all participants via in-person interviews. Medical data such as medical history, previous obstetric history, laboratory parameters such as Hb and glucose values, diagnosis of GDM and PE, and gestational weeks at delivery were extracted from the medical records.

All the investigators at each hospital received standard training before administering the survey. Each completed questionnaire was reviewed by an inspector. Data were coded and entered into a specially designed data software program that automatically flagged out-of-range values and logical errors. All questionnaires were entered independently by two individuals and were then verified by a third person.

Definitions:

1. BMI categories: Pre-pregnancy BMI was calculated as the body weight within the 3 months prior to pregnancy in kilograms divided by height in meters squared (kg/m^2^). Based on the criteria recommended by the China Obesity Task Force of the Chinese Ministry of Health [13], the participants were categorized as follows: underweight: BMI < 18.5 kg/m^2^; normal weight: 18.5 ≤ BMI < 24 kg/m^2^; overweight: 24 ≤ BMI < 28 kg/m^2^; and obese: BMI ≥ 28 kg/m^2^. In this study, we categorized participants into two groups: pre-pregnancy BMI < 24 kg/m^2^ and pre-pregnancy BMI ≥ 24 kg/m^2^.
2. Hb groups: According to the median Hb level in our study (130 g/L) and the definition of anemia during pregnancy (Hb <  110 g/L), we divided the participants into 4 groups: Group 1: Hb <  110 g/L (anemia group); Group 2: 110 g/L ≤ Hb < 130 g/L (reference group); Group 3: 130 g/L ≤ Hb < 150 g/L; and Group 4: Hb ≥ 150 g/L.
3. GDM: The GDM diagnostic criteria followed the newly amended China’s Ministry of Health criteria, which were published in August 2014. These criteria recommended that the diagnosis of GDM should be made when any one 75-g oral glucose tolerance test (OGTT) value met or exceeded 5.1 mmol/L at 0 h, 10.0 mmol/L at 1 h, or 8.5 mmol/L at 2 h when performed between 24 and 28 gestational weeks. Furthermore, an OGTT result of 7.0 mmol/L at 0 h or 11.1 mmol/L at 2 h should be diagnosed with diabetes at any time [14].
4. PE: PE was defined as new-onset hypertension (systolic blood pressure of ≥140 mmHg and/or diastolic blood pressure ≥ 90 mmHg) with new-onset proteinuria (300 mg of protein/day or a urine protein/creatinine ratio of 0.3 mg/dL) after 20 weeks of gestation in a previously normotensive woman [3].
5. Preterm birth: Preterm birth was defined according to the World Health Organization’s criteria as all births before 37 completed weeks or before 259 completed days since the first day of a woman’s last menstrual period [15].

### Statistical analyses

All statistical analyses were performed using the SAS 9.2 statistical software package (Peking University Clinical Research Institute). Figure 4 was created using Stata 9.0 (Peking University Clinical Research Institute). Continuous variables were expressed as the means ± standard deviations, and categorical variables were expressed numerically and as percentages. Differences in the means between groups were assessed using the independent samples t-test and analysis of variance (ANOVA), whereas the Pearson’s chi-square test or Fisher’s exact test (if the variable contained fewer than five measurements) was used for categorical variables. A multivariate binary logistic regression analysis was conducted to evaluate the relative risk by generating the odds ratios (ORs) and 95% confidence intervals (CIs) for GDM, PE, and preterm birth continuously and across different Hb ranges for the overall population and for women with different pre-pregnancy BMI categories. Additionally, Group 2 (110 g/L ≤ Hb < 130 g/L) was defined as the reference group. Models were adjusted for maternal age, pre-pregnancy BMI, gravidity (< 2, ≥ 2), parity (yes, no), education level (≤ 12, > 12), and gestational age at the time of Hb measurement to estimate the ORs for the associations between PE and Hb levels in the first trimester. For the GDM analyses, we further included a family history of diabetes (yes, no) as a confounder, and for the preterm birth analyses, we included GDM and PE as confounders. Additionally, we used restricted cubic spline regressions to model the associations between continuous Hb levels and the risk of GDM, PE, and preterm birth for the entire population and for the different pre-pregnancy BMI subgroups.

## Results

Among the 21,577 eligible women, the mean maternal Hb level in the first trimester was 129.9 g/L. Five hundred eighty-four women (2.7%) had anemia during the first trimester. The baseline characteristics of the total sample population and subgroups stratified by Hb ranges are presented in Table 1. On average, the mean maternal age was 29.8 ± 3.9 years old, 17.5% of the participants had a pre-pregnancy BMI of 24 kg/m^2^ or higher, and most of them had a high education level. A total of 70.4% of the participants were nulliparous, and 11.0% reported a family history of diabetes. Compared with the total population, participants in our study were more likely to have a family history of diabetes (11.0% vs. 9.0%, *p* < 0.001). However, their age, pre-pregnancy BMI, education levels, average monthly income and Hb levels in the first trimester were not significantly different.

**Table 1:** Baseline characteristics of the total sample population and subgroups stratified by hemoglobin ranges

	Total *n* = 21,577	Hb < 110 g/L *n* = 584	110 g/L ≤ Hb < 130 g/L *n* = 9397	130 g/L ≤ Hb < 150 g/L *n* = 11,268	Hb ≥ 150 g/L *n* = 328	*p*-value
Age (years)	29.8 ± 3.9	29.6 ± 4.3	29.7 ± 4.0	29.9 ± 3.7	30.1 ± 4.3	0.005
Pre-pregnancy BMI (kg/m^2^)	21.4 ± 3.3	20.5 ± 4.8	21.0 ± 3.1	21.8 ± 3.4	22.8 ± 4.3	< 0.001
Overweight/Obesity	3783 (17.5)	56 (9.6)	1232 (13.1)	2392 (21.2)	103 (31.4)	< 0.001
Underweight	3330 (14.4)	129 (22.1)	1720 (18.3)	1452 (12.9)	29 (8.8)	< 0.001
Education level (school years) > 12	17,358 (80.4)	399 (68.3)	7358 (78.3)	9341 (82.9)	260 (79.4)	< 0.001
Average monthly income						< 0.001
≥ 5000	8713 (40.4)	255 (43.6)	3787 (40.3)	4552 (40.4)	119 (36.4)	
3000–4999	4287 (19.9)	155 (26.5)	1983 (21.1)	2085 (18.5)	64 (19.4)	
1000–2999	3238 (15.0)	62 (10.7)	1278 (13.6)	1837 (16.3)	61 (18.5)	
< 1000	5339 (24.7)	112 (19.2)	2349 (25.0)	2794 (24.8)	84 (25.7)	
Parity (nulliparous)	12,409 (57.5)	215 (36.9)	4792 (51.0)	7166 (63.6)	236 (71.9)	< 0.001
Family history of DM	2376 (11.0)	27 (4.9)	818 (8.7)	1487 (13.2)	44 (13.5)	< 0.001
Hb levels (g/L)	129.9 ± 9.8	103.2 ± 5.9	122.8 ± 4.9	136.4 ± 4.8	153.9 ± 5.2	< 0.001

Data are represented as the mean ± SD or *n* (%). The *p-*values refer to the overall differences across the Hb groups as derived from ANOVA (for continuous variables) or χ^2^ tests (for categorical variables)

*Abbreviations:* *Hb* hemoglobin, *BMI* body mass index

**Fig. 1:**
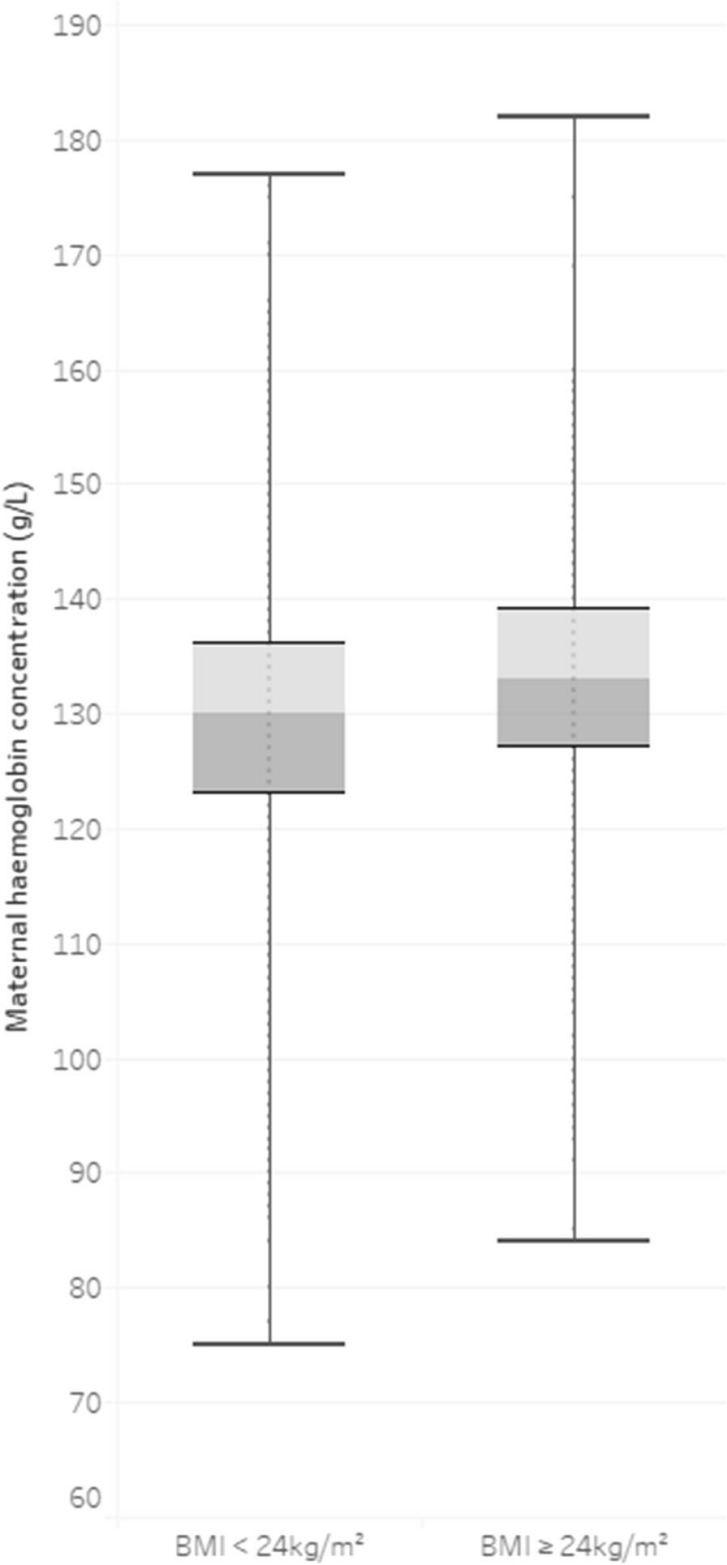
Maternal hemoglobin concentrations in pregnancies with different pre-pregnancy body mass index (p-BMI)

**Fig. 2:**
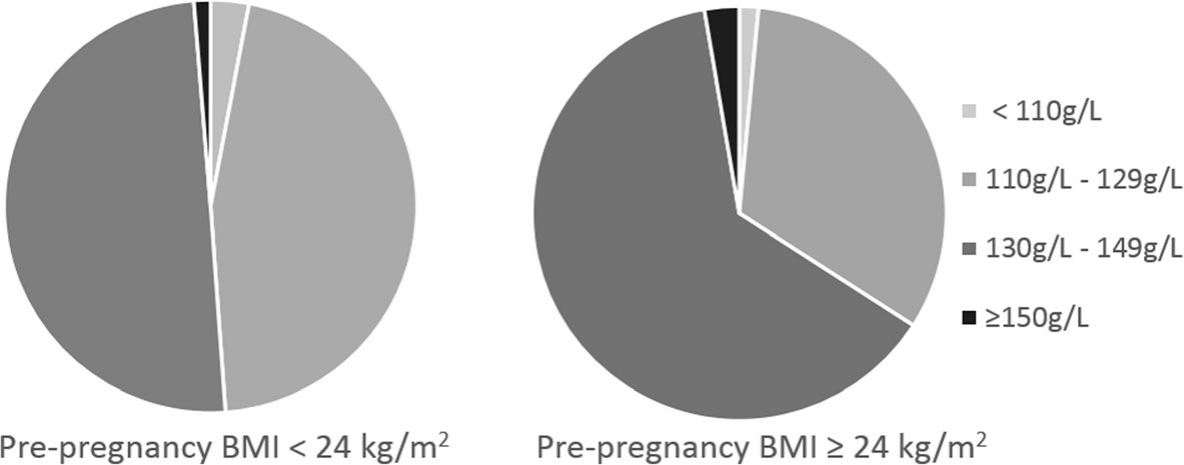
The proportion of hemoglobin concentrations in women with different pre-pregnancy body mass index (p-BMI) categories

Women who were classified as having the highest Hb level in the first trimester were more likely to be older, nulliparous, and have a higher pre-pregnancy BMI and family history of diabetes, whereas women in the lowest Hb level group had the opposite characteristics. For women who were underweight, normal-weight, overweight and obese, early pregnancy Hb levels were 127.8 ± 10.1 g/L, 129.6 ± 9.7 g/L, 132.2 ± 9.5 g/L and 133.4 ± 9.4 g/L, respectively. Furthermore, by dividing the studied population into two subgroups, namely, pre-pregnancy BMI < 24 kg/m^2^ and pre-pregnancy BMI ≥ 24 kg/m^2^, we found that pregnant women in the high BMI group had significantly higher levels of Hb during the first trimester (pre-pregnancy BMI < 24 kg/m^2^ vs. pre-pregnancy BMI ≥ 24 kg/m^2^: 129.3 ± 9.8 g/L vs. 132.6 ± 9.5 g/L; *p* < 0.001) (Fig. 1). The distribution of Hb levels in the first trimester according to BMI can be seen in Fig. 2.

Overall, there were 4337 cases and 284 cases diagnosed with GDM and PE, with an incidence of 20.1 and 1.3%, respectively. There were 1027 preterm births, with an incidence of 4.8%. Compared with the original sample, the incidence of GDM in our study was not significantly different (20.1% vs. 19.7%, *p* = 0.208), whereas the incidence of PE (1.3% vs. 2.2%, *p* < 0.001) and preterm birth (4.8% vs. 7.1%, *p* < 0.001) were significantly lower. Women who subsequently developed GDM and PE had significantly higher Hb level in the first trimester than control subjects, whereas women with preterm deliveries had significantly lower Hb level in the first trimester. However, in women whose pre-pregnancy BMI was < 24 kg/m^2^, the Hb levels were not significantly different between those who subsequently developed PE and those who did not. Furthermore, in women with a pre-pregnancy BMI ≥ 24 kg/m^2^, the Hb levels of those with or without preterm birth were not significantly different (Table 2).

**Table 2:** Maternal hemoglobin levels in women with gestational diabetes mellitus, pre-eclampsia, preterm birth and controls in the first trimester

	Total, *n* = 21,577			Pre-pregnancy BMI < 24 kg/m^2^, *n* = 17,794			Pre-pregnancy BMI ≥ 24 kg/m^2^, *n* = 3783		
	No	Yes	P	No	Yes	P	No	Yes	P
GDM	129.5 ± 9.8, *n* = 17,240	131.3 ± 9.8, *n* = 4337	< 0.001	129.0 ± 9.8, *n* = 14,703	130.5 ± 9.8, *n* = 3091	0.000	132.2 ± 9.4, *n* = 2537	133.2 ± 9.7, *n* = 1246	0.003
PE	129.8 ± 9.8, *n* = 21,293	132.2 ± 10.5, *n* = 284	< 0.001	129.3 ± 9.8, *n* = 17,615	130.6 ± 11.1, *n* = 179	0.062	132.5 ± 9.5, *n* = 3678	134.8 ± 8.8, *n* = 105	0.013
Preterm birth	129.9 ± 9.8, *n* = 20,550	128.9 ± 10.5, *n* = 1027	0.002	129.3 ± 9.8, *n* = 16,988	127.8 ± 10.4, *n* = 806	0.000	132.5 ± 9.4, *n* = 3562	133.0 ± 10.0, *n* = 221	0.501

Data are represented as the mean ± SD

*Abbreviations:* *Hb* hemoglobin, *BMI* mass index, *GDM* gestational diabetes mellitus, *PE* pre-eclampsia

**Fig. 3:**
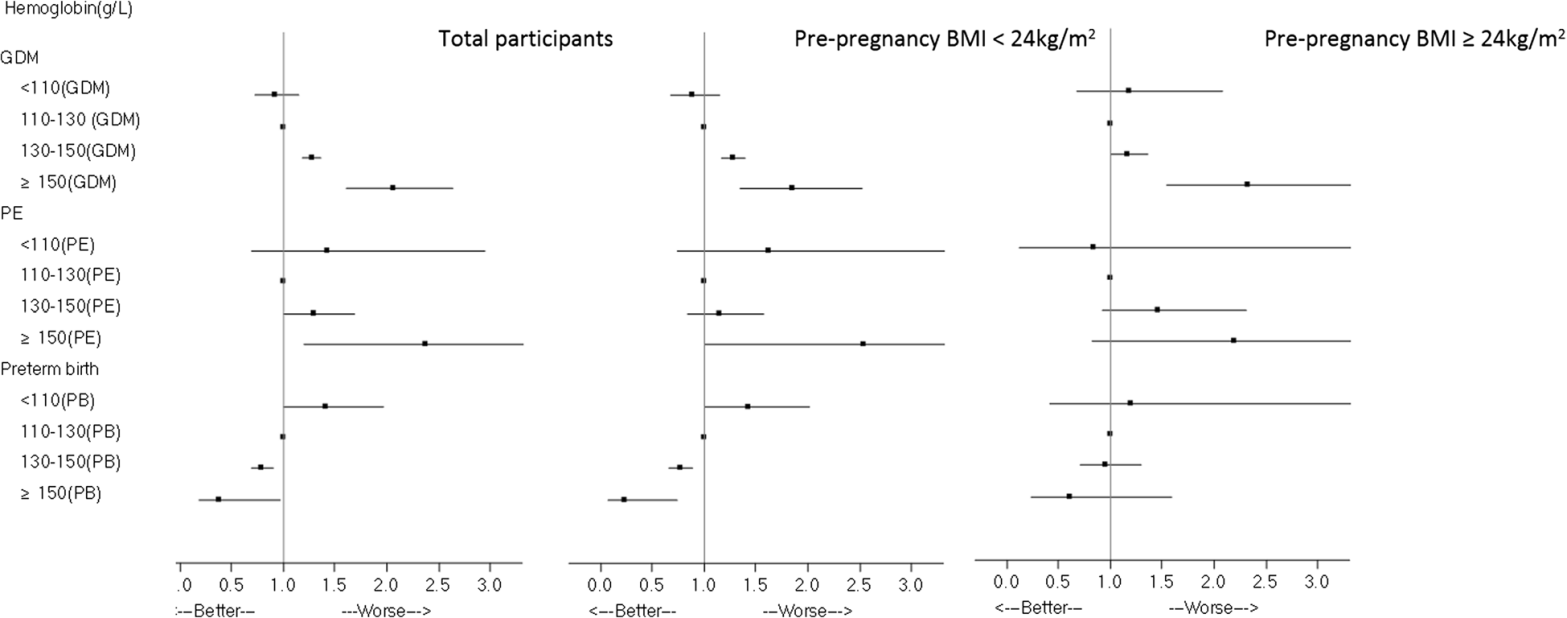
Effects of hemoglobin concentration on GDM, PE and preterm birth during the first trimester

The multivariate logistic regression showed that the Hb levels during the first trimester were significantly and positively associated with the risk of GDM and PE, which were significantly and negatively associated with the risk of preterm birth, even after adjusting for confounders. Overall, the adjusted ORs of GDM and PE increased, and the adjusted ORs of preterm birth decreased across increasing categories of Hb (P-trend for GDM: < 0.001; P-trend for PE: 0.001; P-trend for preterm birth: 0.001). After adjusting for confounders, the ORs comparing the high ranges with the reference range of Hb both demonstrated significantly increased odds of GDM (OR: 1.27 for 130 g/L ≤ Hb < 150 g/L; OR: 2.06 for Hb ≥ 150 g/L) and PE (OR: 1.30 for 130 g/L ≤ Hb < 150 g/L; OR: 2.38 for Hb ≥ 150 g/L). In addition, the ORs of preterm birth (OR: 0.80 for 130 g/L ≤ Hb < 150 g/L; OR: 0.38 for Hb ≥ 150 g/L) were significantly lower for the high ranges than that for the reference range of Hb. Furthermore, a 1.4-fold increased odds of preterm birth was observed when comparing the anemia group with the reference group (see Table 3 and Fig. 3).

**Table 3:** The associations of hemoglobin levels with the risks of gestational diabetes mellitus, pre-eclampsia, and preterm birth

		Total, *n* = 21,577					Pre-pregnancy BMI < 24 kg/m^2^, *n* = 17,794					Pre-pregnancy BMI ≥ 24 kg/m^2^, *n* = 3783				
		No. (%)	Crude OR	Adjusted OR	χ2	P-trend	No. (%)	Crude OR	Adjusted OR	χ2	P-trend	No. (%)	Crude OR	Adjusted OR	χ2	P-trend
GDM	Hemoglobin level (g/L)															
	< 110	92 (15.8)	0.884 (0.703,1.111)	0.913 (0.721, 1.155)	102.066	< 0.001	72 (13.6)	0.763 (0.591, 0.986)	0.880 (0.678, 1.144)	54.94	< 0.001	20 (35.7)	1.246 (0.712, 2.180)	1.178 (0.668, 2.078)	6.52	0.01
	110–129	1641 (17.5)	1	1			1261 (15.4)	1				380 (30.8)	1	1		
	130–149	2493 (22.1)	1.343 (1.253, 1.440)	1.272 (1.182, 1.369)			1699 (19.1)	1.296 (1.196, 1.404)	1.274 (1.171, 1.386)			794 (33.2)	1.114(0.961, 1.292)	1.162(0.998, 1.354)		
	≥150	111 (33.8)	2.418 (1.912, 3.058)	2.063 (1.609, 2.645)			59 (26.2)	1.946 (1.437, 2.635)	1.844(1.344, 2.529)			52 (50.5)	2.286(1.525, 3.427)	2.333(1.537, 3.541)		
	Per-unit increment		1.019 (1.016, 1.023)	1.015 (1.011, 1.018)				1.016 (1.012, 1.020)	1.015(1.011, 1.020)				1.011(1.004, 1.018)	1.013(1.006, 1.021)		
PE	< 110	8 (1.4)	1.304 (0.632, 2.694)	1.422 (0.686, 2.945)	17.626	0.001	7 (1.3)	1.489 (0.682, 3.251)	1.618(0.738, 3.543)	1.22	0.27	1 (1.8)	0.843(0.112, 6.329)	0.843(0.112, 6.345)	4.981	0.095
	110–129	99 (1.1)	1	1			73 (0.9)	1	1			26 (2.1)	1	1		
	130–149	166 (1.5)	1.405 (1.094, 1.804)	1.303 (1.006, 1.689)			94 (1.1)	1.186 (0.873, 1.613)	1.148(0.833, 1.580)			72 (3.0)	1.440(0.914, 2.266)	1.455(0.918, 2.306)		
	≥ 150	11 (3.4)	3.259 (1.731, 6.137)	2.375 (1.203, 4.688)			5 (2.2)	2.519 (1.008, 6.296)	2.535(1.006, 6.390)			6 (5.8)	2.869(1.153, 7.138)	2.195(0.814, 5.919)		
	Per-unit increment		1.026 (1.013, 1.038)	1.020 (1.007, 1.033)				1.015 (0.999, 1.030)	1.013(0.997, 1.030)				1.027(1.006, 1.049)	1.025(1.003, 1.047)		
Preterm birth	< 110	41 (7.0)	1.415 (1.017, 1.969)	1.408 (1.008, 1.967)	15.572	0.001	37 (7.0)	1.436 (1.014, 2.036)	1.419(0.998, 2.019)	19.31	< 0.001	4 (7.1)	1.239(0.436, 3.522)	1.198(0.413, 3.479)	0.10	0.75
	110–129	479 (5.1)	1	1			407 (5.0)	1	1			72 (5.8)	1	1		
	130–149	499 (4.4)	0.863 (0.759, 0.981)	0.795 (0.695, 0.910)			359 (4.0)	0.803 (0.695, 0.929)	0.768(0.660, 0.894)			140 (5.9)	1.002(0.747, 1.342)	0.949(0.699, 1.289)		
	≥ 150	8 (2.4)	0.465 (0.229, 0.944)	0.375 (0.183, 0.768)			3 (1.3)	0.258 (0.082, 0.808)	0.233(0.074, 0.735)			5 (4.9)	0.822(0.324, 2.083)	0.612(0.234, 1.598)		
	Per-unit increment		0.990 (0.984, 0.996)	0.987 (0.980, 0.993)				0.984 (0.977, 0.991)	0.982(0.975, 0.989)				1.005(0.991, 1.020)	1.000(0.986, 1.015)		

*Abbreviations:* *BMI* body mass index, *GDM* gestational diabetes mellitus, *PE* pre-eclampsia, *OR* odds ratio

**Fig. 4:**
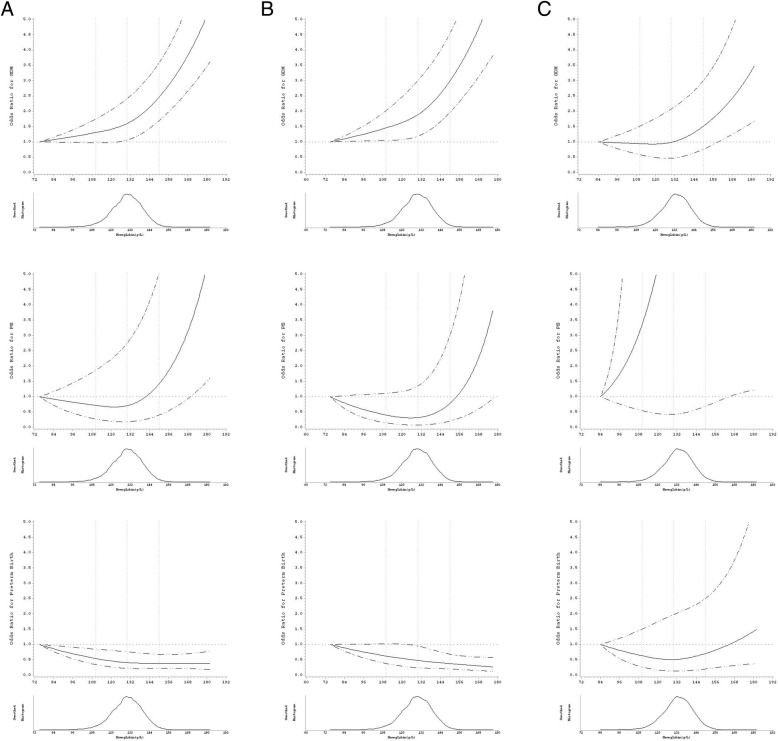
Smoothed histograms and spline plots displaying ORs and 95% CIs describing the association between hemoglobin during early pregnancy and the risk of GDM, PE, and preterm birth among all participants and the two p-BMI subgroups (**a** panel for the total population, **b** panel for women with pre-pregnancy BMI <24kg/m^2^, **c** panel for women with pre-pregnancy BMI <24kg/m^2^

The associations between the Hb level in the first trimester and GDM persisted in the subgroups as defined by the pre-pregnancy BMI. However, the associations between Hb and PE and the associations between Hb and preterm birth were null among the women whose pre-pregnancy BMI was < 24 kg/m^2^ and the women whose pre-pregnancy was BMI ≥ 24 kg/m^2^, respectively. In particular, after adjusting for confounders, Hb levels greater than 130 g/L in the first trimester significantly increased the GDM risk (OR: 1.274 for 130 g/L ≤ Hb < 150 g/L; OR: 1.844 for Hb ≥ 150 g/L), whereas a significantly decreased risk of preterm birth was observed in the women whose pre-pregnancy BMI was < 24 kg/m^2^ (OR: 0.768 for 130 g/L ≤ Hb < 150 g/L; OR: 0.233 for Hb ≥ 150 g/L). Furthermore, the ORs of GDM and preterm birth increased and decreased across increasing ranges of Hb, respectively. When the Hb levels surpassed 150 g/L, the risk of PE in women whose pre-pregnancy BMI was < 24 kg/m^2^ also rose significantly (OR: 2.535 for Hb ≥150 g/L). However, for women whose pre-pregnancy BMI was ≥24 kg/m^2^, only Hb levels higher than 150 g/L were associated with a higher risk of developing GDM (OR: 2.333 for Hb ≥150 g/L). The associations between Hb ≥150 g/L and PE risk were attenuated and became non-significant after adjusting for confounders (Table 3, Fig. 3). Additionally, the continuous association between Hb levels in the first trimester and the risks of GDM, PE and preterm birth in the total population and the BMI subgroups can be seen in Fig. 4.

## Discussion

In this large study of pregnant women, we found that women whose pre-pregnancy BMI was ≥24 kg/m^2^ had significantly higher levels of Hb in the first trimester than women whose pre-pregnancy BMI was < 24 kg/m^2^. Furthermore, women who subsequently developed GDM and PE had significantly higher Hb levels during the first trimester than controls, whereas women with preterm birth had significantly lower Hb levels in the first trimester. After adjusting for potential confounders, Hb levels during the first trimester were significantly and positively associated with the risk of GDM and PE and were significantly and negatively associated with the risk of preterm birth. Moreover, among the women whose pre-pregnancy BMI was < 24 kg/m^2^, Hb levels greater than 130 g/L during the first trimester were associated with a significantly higher GDM risk and lower rates of preterm birth. Furthermore, when the Hb levels exceeded 150 g/L, the risk of PE was significantly elevated. However, among the women whose pre-pregnancy BMI was ≥24 kg/m^2^, only Hb levels higher than 150 g/L increased the risk of GDM.

Similar to our results, an earlier observational study of 730 Chinese pregnant women revealed that high maternal Hb levels (> 130 g/L) in the first trimester were associated with a significantly higher incidence of GDM [16]. As early as 1986, Murphy et al. [17] conducted a study on 44,316 pregnant women and found that women with high Hb levels before 24 weeks of gestation had significantly higher rates of PE. A recently retrospective cohort study of 920 singleton pregnancies indicated that pregnant women with Hb values ≥125 g/L before 14 gestational weeks had a 3.8- and 3.3-fold increased risk of developing PE and GDM, respectively, compared with women with Hb values of 110–124 g/L [10]. Similarly, in the report by Mehrabian and Hosseini [18] on 973 pregnancies during 2011–2012, significantly higher incidences of GDM and PE were found among women who had early pregnancy Hb values ≥125 g/L than those with Hb values < 125 g/L. The OR values of the relative risks were 3.7 (95% CI; 2.2 to 6.4) and 5.4 (95% CI; 2.8 to 10.5), respectively [18]. Our findings are generally in line with those from these studies. Pregnant women in our study with Hb levels ≥130 g/L had an increased risk of GDM and PE, and the association became more significant when the Hb levelss exceeded 150 g/L.

The association observed in this and other studies between Hb levels and the risk of GDM and PE seems biologically plausible. Notably, the results presented in both our study and others [12, 16] showed that women with high Hb levels had higher pre-pregnancy BMI, which suggested that the high Hb levels may be a consequence of their better nutritional status. Furthermore, the high nutritional status may be related to an elevated risk of GDM and PE. Additionally, we hypothesized that high Hb levels may reflect iron overload because iron supplementation in high doses has been confirmed to have a role in the occurrence of GDM [19]. Accumulating evidence has demonstrated that iron is a strong pro-oxidant, and iron overload can increase β-cell oxidative stress, thus causing insulin resistance and impaired glucose metabolism [20].

The mechanism underlying the contribution of Hb to PE may primarily involve high blood viscosity. Hyperviscosity can directly reduce blood flow in the low kinetic force microvasculature, such as the placenta [21]. This may lead to reduced perfusion and oxygenation of placental tissue, therefore exacerbating placental tissue hypoxia as a direct result of low-velocity placental circulation and reduced oxygen supply. Moreover, Hb has a direct role in nitric oxide (NO) regulation and endothelial function. NO is a potent vasodilator and can relax vascular smooth muscle cells. Free Hb can bind and inactive NO, thus leading to vasoconstriction with consequent hypertension [22] and placental ischemia. Furthermore, oxidized Hb could create methemoglobin-derived heme deposits on the vascular endothelium, which in turn directly damage the endothelium or promote atheroma formation via the effect of oxidized low-density lipoproteins [23].

In addition to GDM and PE, our study revealed a significant association between anemia and a high risk of preterm birth. Furthermore, the risk of preterm birth decreased with increasing Hb levels in the first trimester, particularly among women whose pre-pregnancy BMI was < 24 kg/m^2^. Other studies have also confirmed that anemia is an independent risk factor for preterm birth, although studies on Hb levels and the risk of preterm birth are sparse. A retrospective study comparing singleton pregnancies with and without anemia during the first trimester in woman who delivered between 1988 and 2002 showed that compared with non-anemic women, higher rates of preterm birth (< 37 weeks gestation) were found among women with anemia (10.7% vs. 9.0%, respectively, *p* < 0.001). Moreover, maternal anemia was an independent risk factor for preterm birth even after adjusting for confounders (OR = 1.2; 95% CI 1.1–1.2, *p* < 0.001) [24]. At a different period of Hb measurement, an analysis of 295,651 pregnant women found that high preterm rates were observed with Hb levels < 110 g/L, and the relative risk increased across decreasing ranges of Hb. Furthermore, women with Hb levels higher than 145 g/L were also associated with a significantly increased risk of preterm birth (OR: 1.14; 95% CI 1.05, 1.25) [25]. In contrast to the above findings, women with Hb levels > 130 g/L in our study had a significantly decreased risk of preterm birth, and the relative risk decreased across increasing ranges of Hb. However, an international multicenter cross-sectional study of 5690 singleton and nulliparous pregnancies indicated that there was no statistically significant effect of anemia on the risk of preterm birth [26]. In our opinion, variations in population characteristics of the studied participants in the different studies may be the main reason explaining these inconsistent results.

Additionally, several studies have confirmed that iron-deficiency anemia, rather than anemia from other causes, influences preterm birth [27, 28]. Thus, they postulated that iron deficiency may be the cause of preterm birth. However, to date, the exact mechanisms underlying iron deficiency or iron-deficiency anemia and preterm birth have not yet been established. The possible mechanism may involve inadequate transfer of oxygen to the uterus, placenta, and fetus due to a damaged Hb transport capacity caused by iron deficiency.

After we conducted subgroup analyses stratified by pre-pregnancy BMI, we found that the associations of increased Hb with the risk of developing GDM remained in each subgroup. Although a trend towards increasing incidence of PE with elevated Hb levels was only noted in women whose pre-pregnancy BMI was ≥24 kg/m^2^, the protective effects of high Hb levels on preterm birth were seen only in the women whose pre-pregnancy BMI was < 24 kg/m^2^. These results seem to suggest that Hb levels in the first trimester play varied roles on the occurrence of PE and preterm birth in different pre-pregnancy BMI categories. In addition, Hb levels in the first trimester were significantly higher in the women whose pre-pregnancy BMI was ≥24 kg/m^2^ compared to the women whose pre-pregnancy BMI was < 24 kg/m^2^, and their estimated risk Hb level values in the first trimester were 150 g/L and 130 g/L, respectively. Thus, these findings imply that pre-pregnancy BMI should be considered when evaluating Hb levels in the first trimester. However, to date, there are no uniform standards defining a high Hb level.

Our study was a multicenter study and the first to perform a joint analysis of maternal pre-pregnancy BMI and Hb on the occurrence of GDM, PE and preterm birth. The sample size in our study is very large. Additionally, our study was conducted by trained staff, and most of the data were collected from medical records, which ensured the standardization of data collection. Furthermore, the subgroup analyses stratified by pre-pregnancy BMI allowed us to show that pre-pregnancy BMI plays a role on influencing the associations between Hb levels and pregnancy outcomes. However, our study is retrospective, and the recruited pregnant women were from three cities in China. In addition, there are some differences between the original cohort and the final study cohort. Thus, these limitations could introduce bias and limit the generalization of the study findings to all pregnant women in China. Additionally, it should be noted that the sample size of our study may still be insufficient in some of the subgroups, such as the group of women whose Hb levels were < 110 g/L and pre-pregnancy BMI was ≥24 kg/m^2^, which may partially explain the non-significant results. Additionally, the number of participants with PE and preterm birth may also be deficient in our study to evaluate a statistically significant difference due to our exclusion criteria. Recent studies have noted that elevated iron stores may play a role in the development of GDM during pregnancy [19, 29]. However, we do not have data regarding either maternal iron levels or ferritin levels in this study, and we also do not have data regarding supplements taking such as folic acid or iron tablets in the first trimester. Therefore, we could not determine whether the observed anemia was related to iron deficiency, nor make a detailed discussion on the impact of supplements on anemia, nor even perform a joint analysis of Hb levels and iron status in the present study. Furthermore, depending on the results of our study and others, establishing a balance between iron status and Hb levels may be a future challenge and should be revealed through more rigorously designed studies.

## Conclusions

The data shown in our study confirmed that Hb levels during the first trimester play a role in predicting the risk of GDM, PE, and preterm birth. These findings are of clinical and public health importance, since they help clinicians be aware of these complications early in pregnancy other than these syndromes appear overt signs or symptoms later. Thus improving pregnancy outcomes through early intervention to the greatest extent.

## Abbreviations

BMI: Body mass index
CI: Confidence interval
GDM: gestational diabetes mellitus
Hb: Hemoglobin
OGTT: Oral glucose tolerance test
OR: Odds ratio
PE: Pre-eclampsia
